# A tale of two interfaces: vitrivr at the lifelog search challenge

**DOI:** 10.1007/s11042-023-15082-w

**Published:** 2023-04-06

**Authors:** Silvan Heller, Florian Spiess, Heiko Schuldt

**Affiliations:** Department of Mathematics and Computer Science, University of Basel, Basel, Switzerland

**Keywords:** Lifelog retrieval, Lifelog search challenge, Interactive retrieval system evaluation, Virtual reality

## Abstract

The past decades have seen an exponential growth in the amount of data which is produced by individuals. Smartphones which capture images, videos and sensor data have become commonplace, and wearables for fitness and health are growing in popularity. Lifelog retrieval systems aim to aid users in finding and exploring their personal history. We present two systems for lifelog retrieval: vitrivr and vitrivr-VR, which share a common retrieval model and backend for multi-modal multimedia retrieval. They differ in the user interface component, where vitrivr relies on a traditional desktop-based user interface and vitrivr-VR has a Virtual Reality user interface. Their effectiveness is evaluated at the Lifelog Search Challenge 2021, which offers an opportunity for interactive retrieval systems to compete with a focus on textual descriptions of past events. Our results show that the conventional user interface outperformed the VR user interface. However, the format of the evaluation campaign does not provide enough data for a thorough assessment and thus to make robust statements about the difference between the systems. Thus, we conclude by making suggestions for future interactive evaluation campaigns which would enable further insights.

## Introduction

With the increasing availability, affordability and quality of smart, wearable devices, such as smartwatches, fitness trackers, and body cams, the ability of individuals to continuously record their lives in the form of images, videos, audio, and biometric data has increased greatly within the last two decades. Whether to ensure no social-media-worthy situation goes unrecorded, to analyze personal fitness, or simply to keep a digital record of ones experiences, the practice of recording streams of such data, called lifelogging, has become more commonplace with this increased affordability and availability of recording devices. Depending on the frequency and scope of data collection during lifelogging, collections generated in this way, so-called lifelogs, can reach exceptionally large volumes very quickly, making manual management impractical. As such, with the increasing size of lifelogs, it becomes increasingly difficult to analyze and gain insight or retrieve a specific piece of information without efficient computational methods, especially due to the multi-modal nature of lifelog data.

Lifelogs traditionally contain a variety of data with implicit and explicit relations, ranging from videos, to images and structured data such as GPS or heart rate. Lifelog retrieval systems should thus ideally support multiple types of data, and query interfaces supporting different modalities lend themselves well for this use case.

One way to evaluate contributions in the area of multimedia retrieval systems is through interactive retrieval competitions, where different user-system combinations compete with each other. The Lifelog Search Challenge, “modelled on the successful Video Browser Showdown (VBS) [13]” [8], offers such a platform. While there are many different configurations for such evaluations [18], LSC has so far focused on Known-Item Search Tasks, where users are provided textual hints and use their retrieval system to find a target lifelog moment. Thanks to advances in tools [28, 33], these evaluations have also been held online in the past years [13, 27].

Recent evaluation campaigns have also shown that in addition to traditional desktop user interfaces, novel user input methods such as Virtual Reality (VR) can provide new and intuitive retrieval interfaces, while offering competitive performance [9, 13].

In this paper, we present two systems which have participated at LSC in past years: vitrivr^1^ [31] with its conventional desktop user interface, vitrivr-ng and its Virtual Reality counterpart, vitrivr-VR.^2^ Both systems use the same retrieval engine [30] and database [4], but offer different query and interaction methods. vitrivr-VR builds upon the vitrivr system, which has been used in a variety of contexts such as cultural heritage [20, 22, 34], retrieval for speech transcription [39], and video retrieval [11].

Our contribution is threefold: First, we describe in detail how the shared backend of vitrivr and vitrivr-VR handles the lifelog data used at LSC, which features are used and how the retrieval model works. Second, we describe the user interfaces vitrivr-ng and vitrivr-VR for lifelog retrieval, and the implications on the retrieval approach. Third, we compare and analyze the performance of the two interfaces at LSC, providing insights into the differences between the traditional desktop-based system and the VR system for lifelog retrieval. The fact that both systems use a common retrieval engine allows for an interesting comparison. All components of vitrivr and vitrivr-VR are fully open-source.

The rest of this article is structured as follows: In Section 2, we describe the data and retrieval model used by both systems. Section 3 compares the architecture of the two systems, introducing common components and highlighting key differences. The user interfaces are described in Section 4, Section 5 analyses the results of the participation at LSC 2021, and Section 6 concludes.

## Data and retrieval model

In 2021, vitrivr participated for the third time [12, 14, 26] with its traditional desktop interface vitrivr-ng, and vitrivr-VR for the first time [38] in the LSC. Between 2019 and 2021, our conceptual approach to lifelog retrieval has evolved, and this section describes our model for the 2021 iteration in detail.

This section contains our data model, the internal representation of the dataset, in Section 2.1, which is followed by an in-depth look at the retrieval model for multi-modal lifelog retrieval in Section 2.2. The content draws from previous LSC participations [12, 14, 26, 38] and existing work on the conceptual underpinnings of vitrivr [5–7, 15, 23]. It contains some simplifications compared to the actual implementation due to the focus on lifelog retrieval.

### Data model

**Fig. 1:**
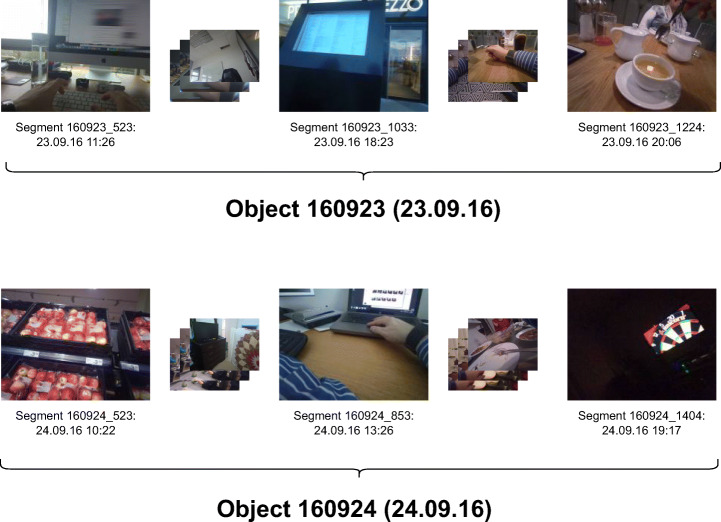
The data model groups segments (individual images) into objects (days). The figure shows selected segments from two consecutive days which are grouped into two different objects. A segment is our smallest unit of retrieval

Fundamentally, we are provided with two different types of data for the LSC: The images captured by the lifeloggers and the metadata accompanying each image^3^. In our data model, the smallest unit of retrieval is called a *segment*, and segments are grouped into *objects*. This distinction comes from the focus of vitrivr on multimedia, and enables us to easily distinguish different result aggregations such as ranking segments individually, or sorting by object. In the context of lifelog retrieval, each individual image is considered a *segment*, and each day is an *object* consisting of all segments within that day. Figure 1 shows an example of two objects (days), each consisting of multiple segments (images).

#### Multimedia data

As our retrieval model is centered on *multimedia* retrieval, there are different types of segments and objects depending on the type of multimedia. For example, in video retrieval the original videos are represented as objects, on which we perform shot segmentation to generate partial video sequences (*shots*) which are represented as segments. At the LSC we are provided with individual images with the associated timestamp of capture, which we use as our smallest unit of retrieval, and thus consider each image a segment. Different summarizations are possible, such as by week, month, or semantic events. We have chosen to select a day as our abstraction level, which is illustrated in Fig. 1.

We assign to each object *o*, i.e., each day, a unique identifier and store the path *path* where all images for that day are located.^4^ No content is stored for an object, as the actual content (images) is stored per segment. All segments for a given object are stored in a single folder, which is stored as *path*.

$$ o := \left<objectId, path\right> $$

Since each segment *s* is part of an object, we generate a unique identifier per segment and store it alongside the objectId, the sequence number (i.e., its index within a day), and its timestamp. The actual image is stored in the file system and fetched on demand. For example, the segment with the tea cup in Fig. 1 has the sequence number 1224, is taken at 20:06 and belongs to the object 160923 (all images from the 23^*rd*^ of September 2016)

$$ s := \left<segmentId, objectId, sequenceNum, timestamp, path\right> $$

#### Metadata

We separate provided metadata into two categories: metadata which is used for Boolean retrieval, and metadata which is used for content-based retrieval (e.g., text).

##### Metadata for boolean retrieval

The metadata model looks the same for objects and segments. Given the unique identifier *id* of either one of the two, a metadata tuple *m* is defined as follows:

$$ m := \left<id, domain, key, value\right> \text{ with } id \in \left\{segmentId, objectId\right\} $$

The most commonly used *domain* values are *technical* e.g., for resolution, *provided* for metadata which is provided by external sources, but this also allows to add e.g., *exif* as a domain for metadata which comes with an image. An example tuple storing the timezone for our example segment from Fig. 1 could thus be $\left <\text {160923\_1224}, \text { provided}, \text { timezone}, \text { Europe/Dublin}\right >$.

##### Metadata for content-based retrieval

Provided annotations such as OCR and textual descriptions are used during retrieval along with other features which we extract ourselves, which is discussed in the next section.

### Retrieval model

At its core, our retrieval model is based on a *late fusion* of different features, which each consider different aspects of a query. Each feature is responsible for returning the best matching segments for a query, and the scores are fused together in a second step. More formally, each feature produces a list of *scored segments* $\hat {s} = \left <segmentid, score\right >$ containing a segment identifier and the score for said segment (scores are bound, 0 ≤ *score* ≤ 1).

In the following, we will first introduce the features which are available, then how they can be queried, and how different query modalities can be combined to generate a single ranked list. Afterwards, extensions which were made to enable queries with temporal context are briefly described.

#### Features for lifelog images

Our retrieval engine implements a variety of features for different types of media [6, 23]. In this section we briefly list the features used for lifelog retrieval.

**Fig. 2:**
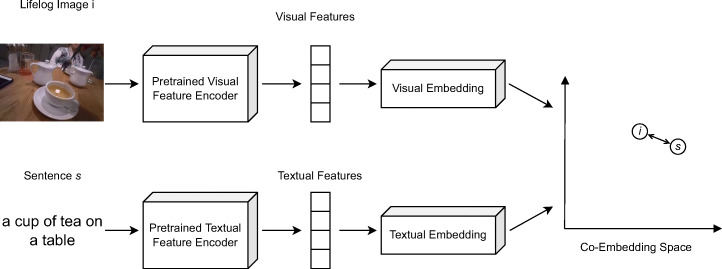
Conceptual overview of our visual-text co-embedding method (adapted from [37])
Boolean Metadata Retrieval: We focus on two types of metadata queries: *range* for e.g., hour and *option* for e.g., day or timezone. Range queries get transformed to simple *BETWEEN* request to the database, and for options we support various Boolean operators such as =, ≤,*or* ≥.
Concept Classification / Tag Retrieval: In contrast to the visual-text co-embedding feature, which takes free text input, this feature assumes a pre-defined set of concepts (or *tags*) have been classified. In the past, we have used 3DConvNets [42] or external APIs [32]. For LSC 2021, we have used the provided annotations and their score which is used by our tag retrieval feature. More formally, given a set of user-provided tags $T=\left <t_{1}, t_{2}, \ldots , t_{k}\right >$, we retrieve for every tag all matching segments. Given a relevance score per segment-tag combination *rs*(*s*,*t*), the score of a segment is computed by summing all scores and normalizing:
  $$ sc_{\text{tag}}(s,T) = \frac{{\sum}_{i=1}^{k}rs(s,t_{i})}{k} $$
Geospatial NNS: We support simple queries in the form “near this point”, meaning the retrieval engine takes as input a single point of the coordinate system as a query $cq=\left <lat_{cq},lon_{cq}\right >$. This is then compared to the coordinates of each segment, $cs=\left <lat_{cs},lon_{cs}\right >$, and compared using the Haversine distance *hav*. Transformation to a similarity score is done using hyperbolic correspondence with a configurable value *divisor* which determines the distance at which the score should be 0.5^5^
  $$ sc_{\text{geo}}(cq,cs) = \max \left( 0,1-\frac{1}{1+\frac{\text{hav}(cq, cs)}{divisor}} \right) $$
OCR: Over the years, we have experimented with different OCR features such as a combination of EAST [46] and CRNN [35] in 2019 [32], but have not used OCR in 2020 and 2021. For 2022, we will use HyText [41].

Visual-Text Co-Embedding: Recent evaluations have shown that features transforming text and images into a joint embedding space are very competitive in interactive video retrieval [13, 19], and the popularity of such embeddings for systems participating at LSC [1] show that they are also considered essential for state-of-the-art systems supporting lifelog retrieval. We use a very simple yet effective visual-text co-embedding based on pre-trained visual and textual feature encoders and embedding networks trained by us [37]. Figure 2 shows a conceptual overview of this feature. The pre-trained feature encoders used are the InceptionResNetV2 [40] for visual features and the Universal Sentence Encoder [3] for textual features. Our models were trained on a mixture of captioned image and video data from Flickr30k [45], Microsoft COCO [17], MSR-VTT [44], TextCaps [36], TGIF [16], and VaTeX [43]. For regularization, the output vectors are normalized to the 256 dimensional hypersphere. During retrieval, the similarity between a query vector *qv* and a known segment vector *sv* is calculated as the Euclidean distance between the two vectors transformed to a similarity score with a linear correspondence function with a factor of 2:
  $$ sc_{\text{vtce}}(qv_{i},sv_{i}) = \max\left( 0,1-\frac{\sqrt{{\sum}_{i=1}^{256}(qv_{i}-sv_{i})^{2}}}{2}\right) $$

#### Query model

**Fig. 3:**
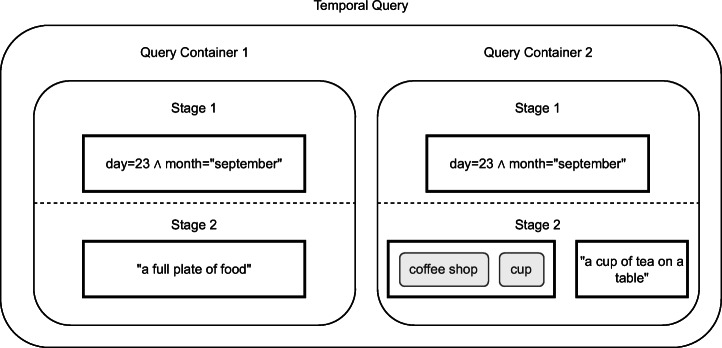
The query model allows users to specify a sequence of queries. In this example, a user is looking for images from the 23^*rd*^ of September, where there is first a full plate of food and then a cup of tea on a table. The second query container also has the tag modality

**Fig. 4:**
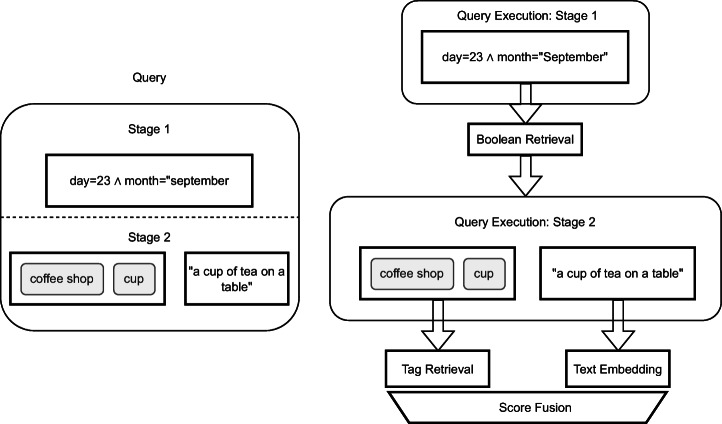
Example of a staged query where the search space for the content-based features (tag and visual-text co-embedding) is limited to elements on the 23^*rd*^ of September. The results of the tag and text feature are merged in the front-end to generate the final result

Our retrieval model allows combining different query modalities such as text and Boolean retrieval (and thus features) in different ways. We show an example query in Fig. 3. At the highest level, users can specify temporal sequences using multiple *query containers*, such as first seeing “a full plate of food” and then “a cup of tea on a table”. Each query container represents an information need for a single segment. We enable users to restrict the search space of a feature by the output of a previous one, e.g., by searching only within the images of the 23^*rd*^ of September as done in both containers. We call this a *staged query* [15]. The simplest combination of modalities having them on the same level with equal importance, as done i.e., in Stage 2 of the query in Fig. 4.

#### Query execution model

We will now discuss how the results of the individual features are combined given the different options from the query model, moving from simple to complex fusion. We show an example of staged query execution in Fig. 4.

##### Feature fusion

If multiple modalities are present in one stage, we offer a configurable choice between max-pooling and average-pooling, i.e., given a list of similarity scores (*sc*_1_,*sc*_2_,…,*sc*_*j*_) for a segment, either $score=\max \limits (sc_{1}, sc_{2}, \ldots , sc_{j})$ or *score* = avg(*sc*_1_,*sc*_2_,…,*sc*_*j*_). For LSC 2021, vitrivr and vitrivr-VR have both used average-pooling. In Fig. 4, the results of the tag and visual-text co-embedding feature are fused together. Features are fused in the front-end which allows users to weigh features differently without re-executing a query.

##### Staged queries

For lifelog retrieval queries, there are often hard binary constraints provided, such as the day of the week or a timeframe (e.g., evening). In those scenarios, it makes sense to limit the search space for similarity-based queries in advance. Our database layer supports this functionality by taking a list of allowed ids as an optional query parameter. In the example of Fig. 4, the user specifies the Boolean query for day and month as a first stage. Only the ids for this feature are returned, the features in the second stage start retrieving, as they receive the list of allowed ids in addition to the user-specified query. This necessarily takes place in the backend, but the results are still returned per feature, to allow fusion in the front-end.

##### Temporal queries in lifelog retrieval

Starting in 2020, we have experimented with different ways to allow users to specify the temporal context of their queries, and different algorithms and architectures for these queries. This has been identified as an important component for interactive multimedia retrieval system in the analysis of interactive retrieval competitions, e.g., in VBS 2020 “The results reveal that the top two systems mostly relied on temporal queries before a correct frame was identified” [19] and 2021 “[...] almost all top performing systems [...] enable specification of temporal context in queries” [13]. Our current approach allows users to specify a *sequence of queries*, each describing a desired element. An individual query in this sequence is called *Query Container*.

The results of these individual queries are then fused together to rank objects higher which fit the specified query sequence. Our approach for video retrieval is described in detail in [10], and is adopted for lifelog retrieval with minor modifications.

Our current model has several drawbacks when applied to lifelog retrieval, such as the fact that object-level constraints such as the date have to be specified per query container. Additionally, we have yet to perform a thorough investigation into the performance of our algorithms in the context of lifelog retrieval.

## Architecture

**Fig. 5:**
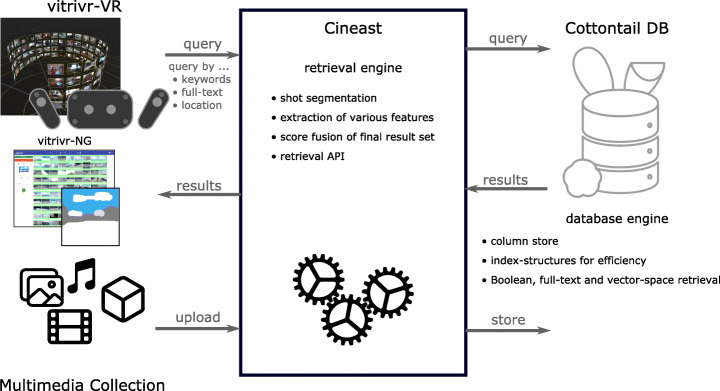
Architecture of both systems, with the shared components of the retrieval engine and the database. Adopted from [37]

As mentioned previously, both vitrivr and vitrivr-VR share a common retrieval engine called Cineast [29, 30], and rely on a specialized database for multimedia and Boolean retrieval, Cottontail DB [4]. Figure 5 shows a high-level view of the architecture of the two systems.

In this section, we will briefly discuss how the different components are related to each other the separation of concerns between the layers. This builds the foundation for Section 4, where we describe the user interfaces in detail.

Database: Cottontail DB supports vector-based similarity search, text retrieval and simple Boolean retrieval. Since it does not offer joins, metadata and segment/object information lookup is done by the retrieval engine. As discussed in previous section, all functionality is used, e.g., visual-text co-embedding uses vector-based similarity search and Boolean retrieval is used for metadata.
Retrieval Engine: Cineast is responsible for feature extraction ahead of the competition and provides a GRPC, Websocket and RESTful API for query execution.

Due to technical reasons, the systems use different API methods. vitrivr uses the Websocket API of Cineast, whereas vitrivr-VR uses the RESTful API, but the underlying retrieval model is the same.

vitrivr has a web-based user interface called vitrivr-ng which is implemented in Typescript^6^ and uses Angular^7^. First introduced in [6], it also has served as the basis for LSC participations by other teams [24, 25]. vitrivr-VR is implemented in Unity and uses OpenXR.

## A tale of two interfaces

Having discussed the conceptual retrieval model which is shared between the two systems and their common architectural components, we now turn to their main defining difference: the user interface. Both user interfaces allow query formulation with different modalities and result presentation, with the main differences being that vitrivr-VR does not support late filters nor staged or temporal queries.

In this section, we will cover each user interaction aspect in one of the following subsections. While sketch queries are supported by both systems, they were not used in the LSC context and therefore are not discussed here.

**Fig. 6:**
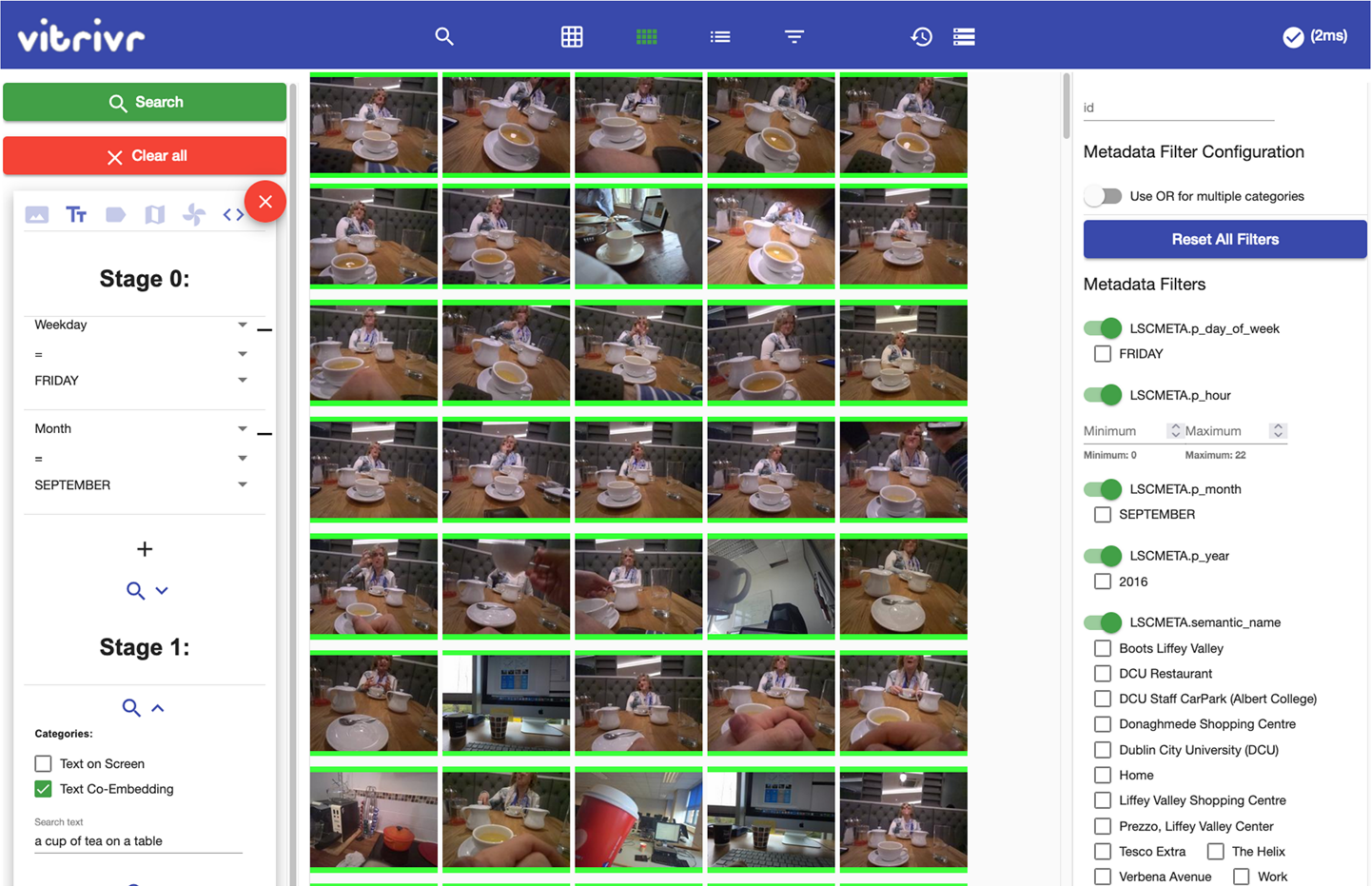
Full overview of the vitrivr-ng user interface. On the left side, users can formulate queries. The middle part shows results, and on the right side, filters can be applied

**Fig. 7:**
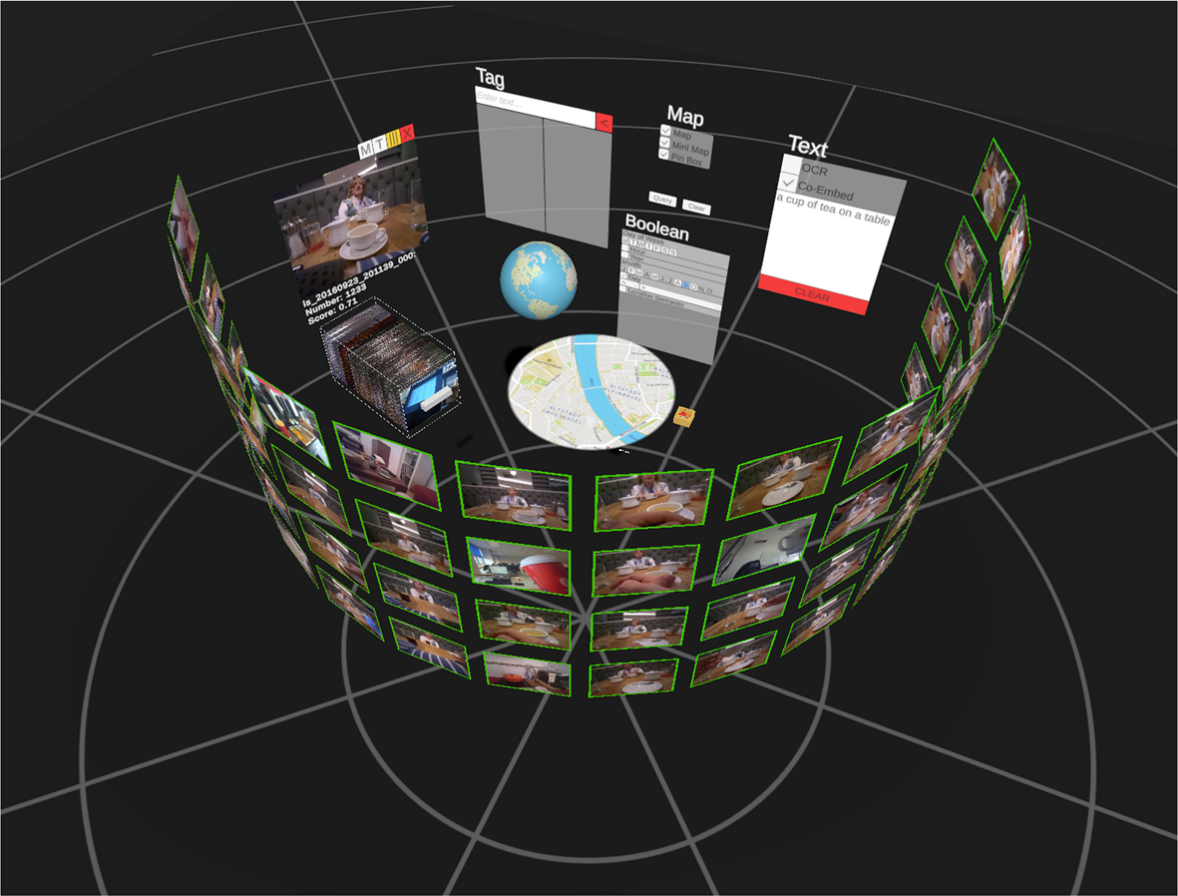
Overview of vitrivr-VR showing the modular query formulation UI surrounded by the cylindrical results display and one result shown in detail view with the temporally neighboring images shown in the form of a drawer of images below it. Due to the inherent nature of made-for-VR content, it is not possible to clearly depict all aspects of vitrivr-VR in a single 2D representation

**Fig. 8:**
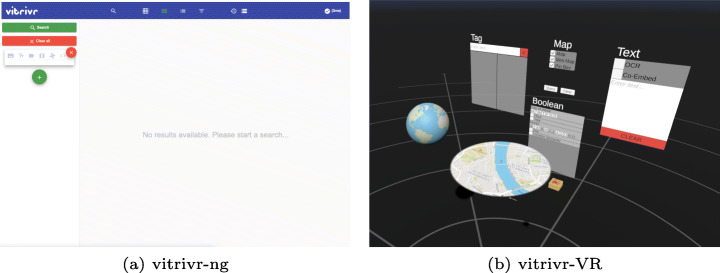
Side-by-side comparison of the entrypoints of the two systems

In Fig. 6 and 7, we show the two user interfaces in action, with a query already formulated and results visible. In vitrivr-ng, query formulation happens on the left side of the screen, and results are displayed in the center. Different result views can be toggled in the header. vitrivr-VR takes a more free-form and modular approach to the user interface in an attempt to make use of additional freedom in VR. The query formulation interface is separated into panels by modality. Each of these interfaces can be grabbed by the user and rearranged in virtual space. In vitrivr-VR, query results are displayed in a horizontally scrollable display that wraps cylindrically around the user. Figure 8 shows a comparison of the entrypoints of the two systems.

### Query formulation

**Fig. 9:**
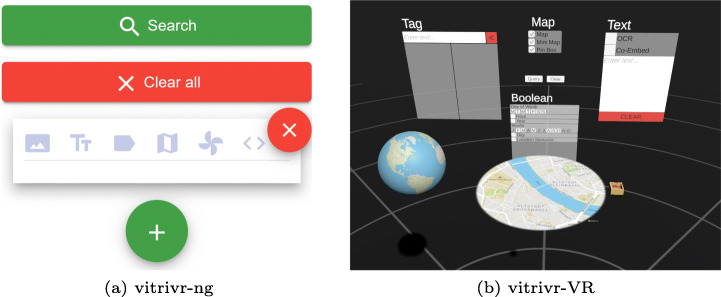
Side-by-side comparison of the query formulation view

**Fig. 10:**
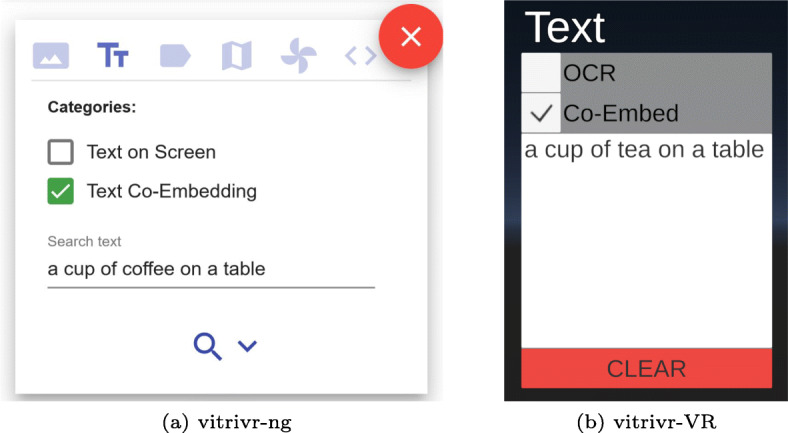
Side-by-side comparison of the textual query formulation modality

In Fig. 9, we show a comparison of the query formulation views. In vitrivr-ng, all modalities can be toggled. Additionally, new query containers can be added by clicking on the green plus button, which allows adding temporal context. This enables users to specify temporal sequences in their query. When multiple modalities are used within the same query container, they can be individually pushed to a later stage by clicking on the downward arrow next to the magnifying glass visible in Fig. 10a.

In the current version of vitrivr-VR, staged and temporal queries are not yet available, making query formulation less expressive, but also much simpler. All available query modalities are available as individual interfaces in virtual space and are automatically used for subsequent queries as soon as the user inputs data. The text term input, for example, is used for queries as long as at least one text category has been selected and the user has input text in the text field.

As highlighted in previous sections, our model for lifelog retrieval allows a seamless combination of different modalities. The query formulation for those modalities will be described in the following.

#### Textual queries

Both interfaces allow textual queries for OCR or visual-text co-embeddings, and a comparison is shown in Fig. 10. vitrivr-ng offers traditional text input, where boxes can be checked depending on the desired feature (e.g., OCR, textual embedding). vitrivr-VR offers a very similar interface in VR, where different feature categories can be toggled via a set of check-boxes attached to a text field. Text input in vitrivr-VR is facilitated through speech-to-text and a virtual keyboard. The speech-to-text solution, based on DeepSpeech^8^, is particularly useful to quickly enter long scene descriptions for the visual-text co-embedding, while the virtual keyboard allows the input of hard-to-pronounce words and precise corrections.

#### Boolean queries

**Fig. 11:**
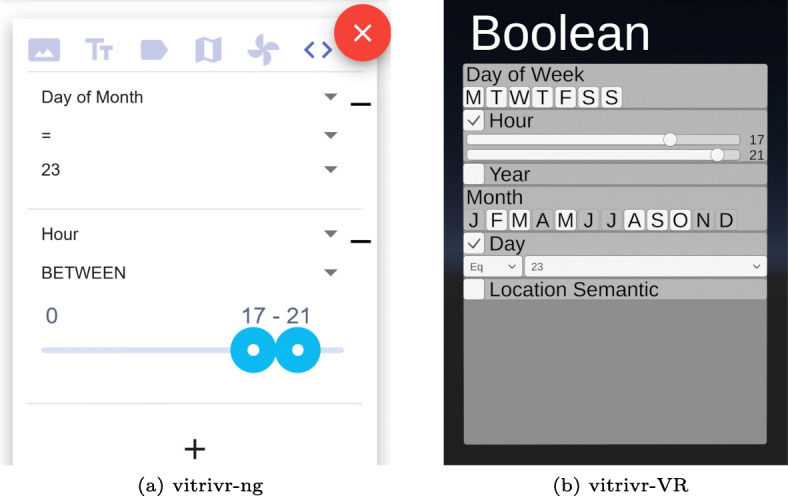
Side-by-side comparison of the Boolean query formulation view

vitrivr-ng mainly supports two types of Boolean queries, shown in Fig. 11a: simple dropdowns, either with a provided list of options or dynamically generated based on distinct column values, or range queries for e.g., hour of the day. Additionally, the same query modality offers text retrieval e.g., for segment ids as a convenience feature.

vitrivr-VR supports the same two types of Boolean queries through sliders for ranges and drop downs for single selections, but also supports multiple-response style selection e.g., to select the days of the week. This multiple-response style selection results in a Boolean expression specifying a segment’s membership in the selected set of options. As seen in the month selection in Fig. 11b, multiple-response style selections indicate if data fitting a certain value even exist by disabling the options that do not represent any data.

#### Geographical queries

**Fig. 12:**
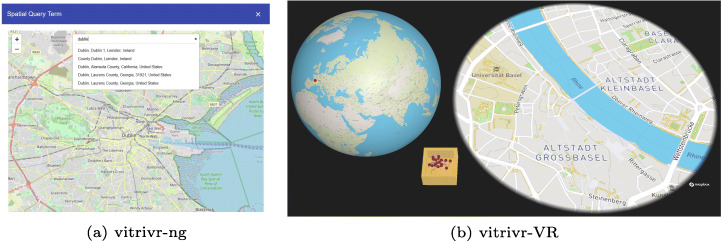
Side-by-side comparison of the geospatial query formulation view

For information needs with a spatial context, vitrivr-ng supports the simple use case of putting a pin on the map and searching for segments in proximity. This is implemented using Leaflet^9^ and OpenStreetMap^10^, and the leaflet-geosearch package^11^ is used for location lookup independent of the dataset, i.e., searching for “Dublin”. Figure 12 shows the query formulation view of both systems.

In vitrivr-VR the geospatial query interface consist of three parts: a globe-like *mini-map*, a flat *detail-map* and a *pin-box*. Geospatial queries are formulated by grabbing a pin from the pin-box in virtual space and sticking it into the detail-map. To specify locations outside of the detail-map’s default view, it can be dragged with one hand to pan the map and zoomed through a pinching gesture using both hands. To quickly change the location displayed by the detail-map without having to zoom the map out and in again, a pin can be placed on the globe mini-map, which immediately sets the detail-map to focus on the corresponding location. All parts of the geospatial query interface can be grabbed and placed anywhere in virtual space and even disabled, allowing users to customize the query interface to their individual needs.

### Result presentation

Having formulated the queries, the retrieval engine returns results per segment and feature^12^, allowing the user interface to determine the result view and final ranking.

Both interfaces allow two different result presentation views: A ranked segment list, shown in Section 4.2.1, and views which groups segments together and aggregates their score, shown in Section 4.2.2. The UI for inspecting segments is shown afterwards in Section 4.2.3.

The result presentation of vitrivr-ng and vitrivr-VR differs in the details of presentation, but there are also some bigger functional differences between the two interfaces. While the result displays offered by vitrivr-ng are more interactive and allow additional configuration through late filtering, only the result set of the most recent query can be viewed. vitrivr-VR, by comparison, does not support late filtering, but preserves the results from previous queries, allowing users to return to previous result set including their scrolling position within the results. Furthermore, by allowing results to be pulled out of a results display and placed anywhere in virtual space, vitrivr-VR allows specific results to be kept independently from the query results from which they were taken, and to be used as reference or for comparison while viewing results from a different query.

#### Ranked segment list

**Fig. 13:**
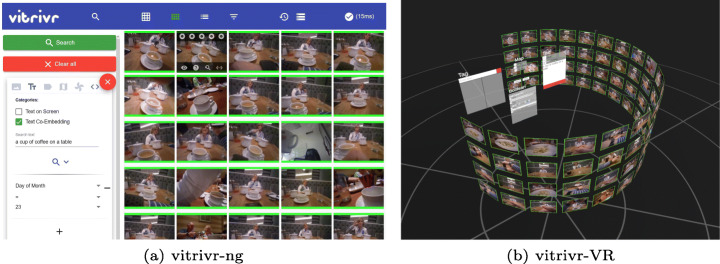
Ranked segment list result display. Images from different days and events are scored individually, and ranked by score

The default view for both interfaces orders individual segments (that is, individual images in our context) by their fused score. Shown in Fig. 13, this is a classic 2D-grid in vitrivr-ng and a horizontally scrolling 2D-grid that wraps cylindrically around the user in vitrivr-VR. While additional results can be viewed in vitrivr-ng via vertical scrolling, in vitrivr-VR results scrolled beyond 360 degrees around the user are hidden from the display and replaced with new results in decreasing order of similarity score. This interaction is performed through regular scrolling in vitrivr-ng, while it is performed through the primary touchpad or joystick in vitrivr-VR.

#### Segment aggregation views

**Fig. 14:**
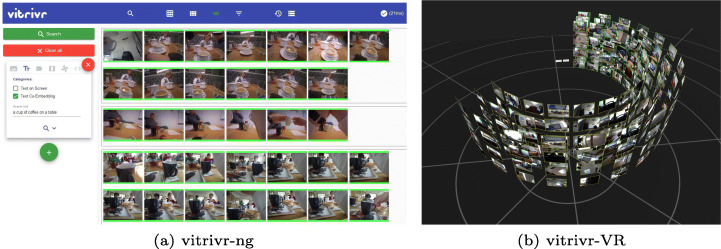
Object-based result display in vitrivr-VR in a cylindrical grid (query formulation UI disabled for clarity) and in vitrivr-ng as a list, with object (days) separated by double grey lines

Both interfaces offer a result view which is based on object scores, which is a score for a given day in the LSC context. The two interfaces differ both in the way these object scores are calculated and how they are displayed. In vitrivr-ng, objects are scored through average-pooling of the segment scores, i.e., given all scored segments $\hat {S}_{o} = (\hat {s}_{o}^{1}, \hat {s}_{o}^{2}, \ldots , \hat {s}_{o}^{n})$ for an object *o*, the score is determined as follows:

$$ score_{o} = \frac{{\sum}_{i=1}^{n} \text{score}\left( \hat{s}_{o}^{i}\right)}{n} $$

vitrivr-VR uses the maximum score of all segments to rank objects in its segment aggregation results view. In this results view, each object (in the case of the LSC dataset, each day) is assigned a position in the cylindrical grid, ordered by the maximum score of the segments it contains. A fixed number of segments of this object is then shown in this position, one behind the other, ordered by segment score. These views are shown in Fig. 14.

**Fig. 15:**
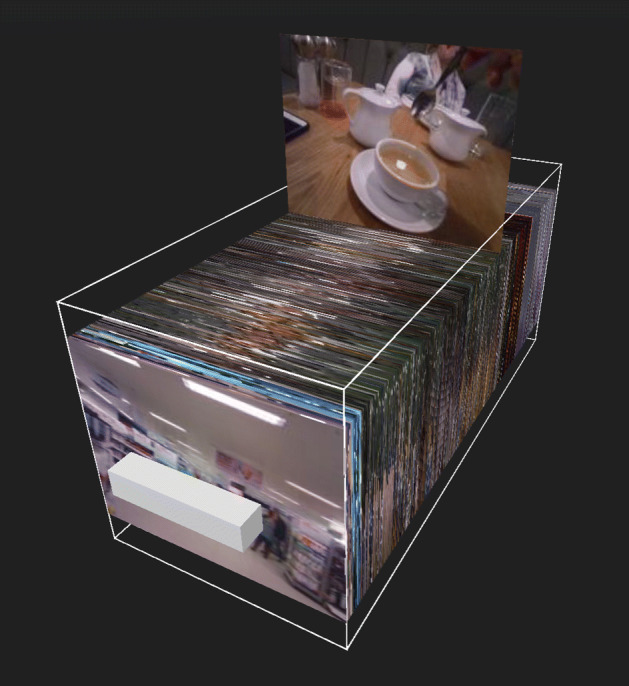
The drawer view of the segments of a day in chronological order

Both vitrivr-ng and vitrivr-VR support different additional segment aggregation views. vitrivr-VR provides an additional drawer view, showing the segments of an object neighboring the selected segment in chronological order inside a virtual box, which can be moved by the user. This view is shown in Fig. 15. By moving their hand into this virtual box and hovering over a segment image, users can take a closer look at individual segments, which are then shown above the box. By quickly moving their hand through this virtual box, users can ‘riffle’ through the segments, producing an effect similar to that of a flip-book. The virtual box containing the segment images can be moved around. Moreover, users can also elongate the box to simplify segment selection, by grabbing and pulling the handle, similar to the action of pulling out a drawer.

**Fig. 16:**
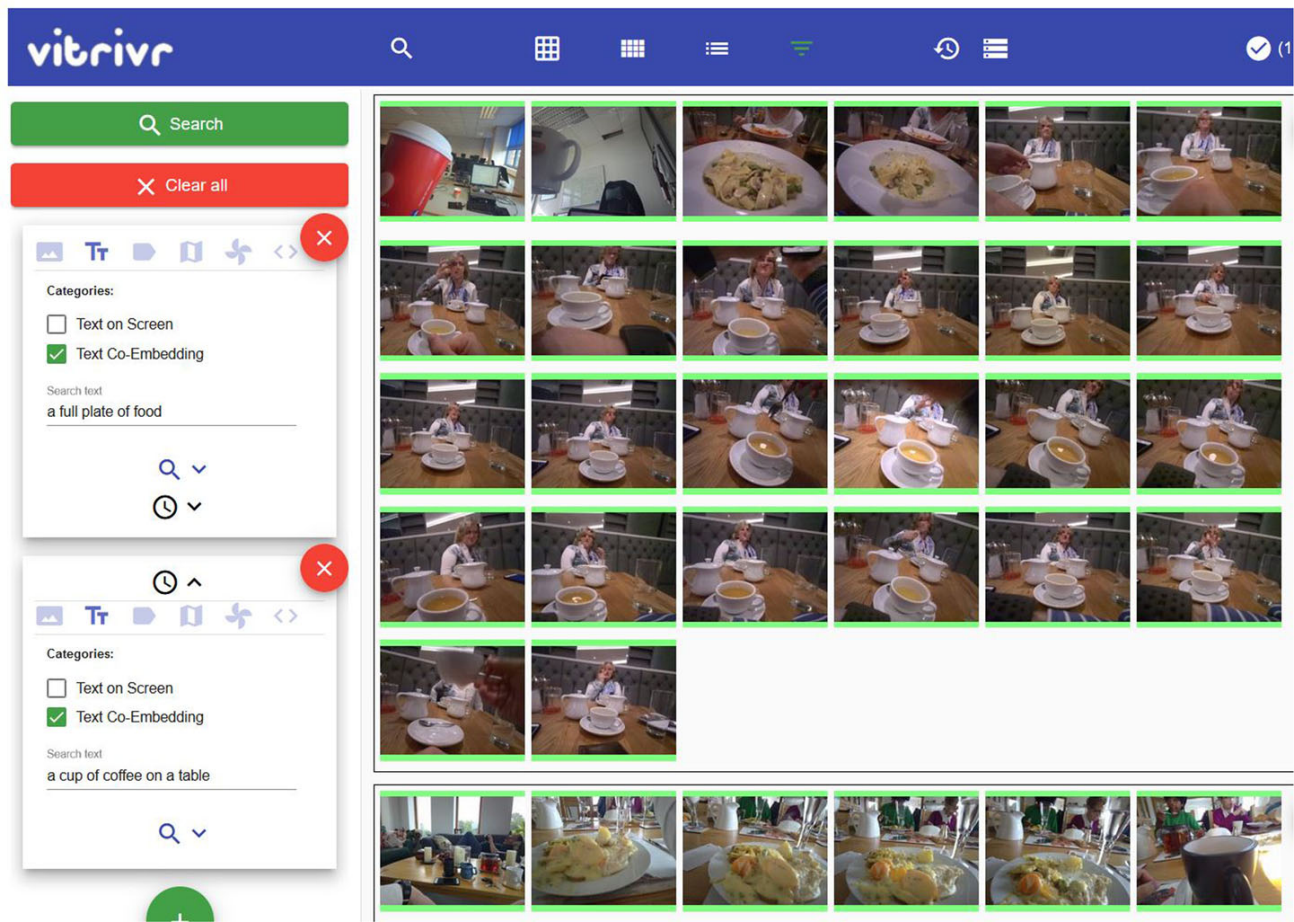
vitrivr-ng also allows specifying temporal context, and groups segments together which match the order of the query

In addition to its regular segment aggregation view, vitrivr-ng offers a temporal scoring view, which computes sequences of segments which match the user-specified query sequence as discussed previously. In our context, this means that for a given day, there can be multiple sequences each with their individual score. The result view shown in Fig. 16 looks similar to that for objects, which makes sense given that it can also be looked at as a different way of aggregating segment scores.

#### Segment inspection

**Fig. 17:**
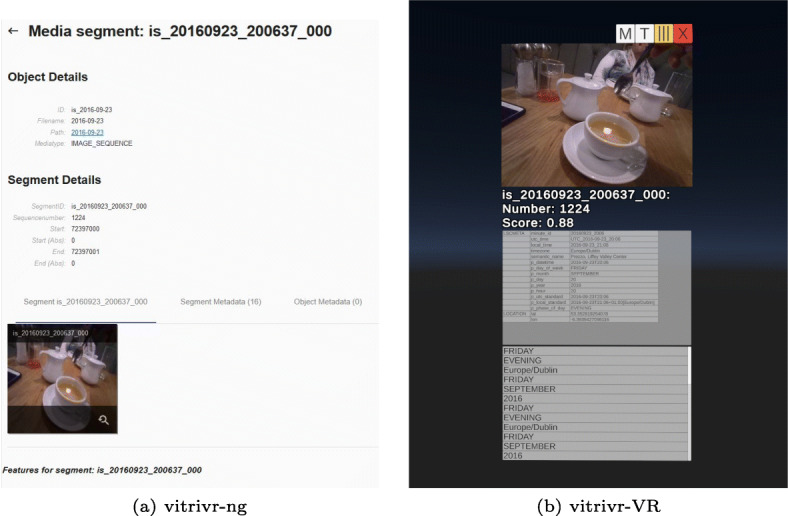
Segment inspection which allows looking at metadata

If a user is interested in closely inspecting a segment to see whether it matches their information need, both user interfaces offer the ability to look at accompanying metadata, and vitrivr-ngalso shows extracted features. We show a side-by-side comparison of the views in Fig. 17.

#### Filtering of result sets

Given that in LSC, new hints appear over time, it is useful to have front-end filters which avoid reformulation and execution of a query. vitrivr-ng has added this functionality specifically for LSC. Two types of late filters are supported: checkboxes which are dynamically generated based on the available metadata, and range filters for e.g., time of day.

## LSC 2021 result analysis

Our analysis focused on the results of LSC 2021, both due to the scope of the special issue and the fact that this was the first year vitrivr-VR participated, which allows for a comparison of the two participants. We use logs provided by the competition server DRES [28], and post-process them into an SQLite database which makes for easier analysis. The final database was provided to all teams. Our analysis is based on the methodology of the VBS 2021 analysis [13]. We first compare the relative performance of the two systems through analysis of the solved tasks, then analyze and discuss indicators of browsing efficiency, and finally provide recommendations for improving the data collection with the goal of enabling more in-depth analyses in the future. The data and results show that limited robust conclusions can be drawn, which is why in Section 5.3 we discuss recommendations for future iterations of LSC and similar interactive evaluations.

### Solved tasks

Table 1 shows an overview of correctly solved tasks by the two teams. Even though both systems shared the same features and retrieval model, the operator of vitrivr solved three tasks that the operator of vitrivr-VR did not solve, but vitrivr-VR also solved one tasks which was not solved by vitrivr. Due to the similarities between system capabilities, these differences in solved tasks can be most likely attributed to operator behaviour, such as query formulation and browsing strategies. While the low number of samples we can analyze is partly because both systems together were only able to solve nine of the twenty-four tasks, LSC could benefit from having multiple independent operators for each system. Such an increase in sample size was already shown to increase the statistical robustness of interactive evaluations [27].

**Table 1:** Tasks which were solved by at least one of the two systems, together with the time in seconds until correct submission

Task	vitrivr	vitrivr-VR
01	168	**97**
04	**36**	60
06	**101**	282
07	**260**	–
08	**166**	–
13	**194**	–
15	94	**47**
24	294	**278**
23	–	**53**

The faster system has its time highlighted in bold

Due to the low number of data samples, it is difficult to make any conclusive statistical statements, however the data we do have indicates, that there are no clear system specific patterns with regards to task solve time. Neither system-operator combination clearly outperforms the other with regards to the time to solve a task, with only a very slight tendency of vitrivr solving more tasks than vitrivr-VR.

### Browsing efficiency

Our main tool to investigate the difference between the two systems is the result logs that both systems submitted to DRES. This allows us to compare the browsing efficiency between the two systems in two key aspects: We can compare the time it takes between the appearance of a correct item in the result view until the operator submits it, and if there were items in the result views that would have been correct, but were not submitted.

**Fig. 18:**
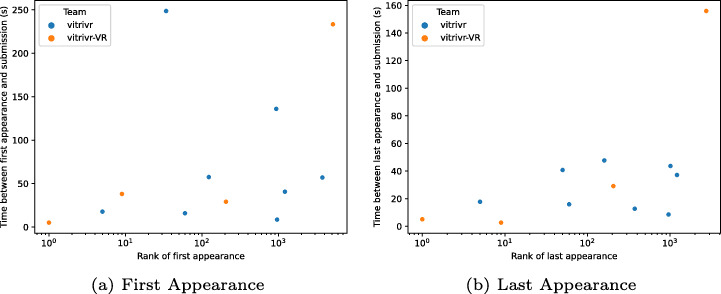
Time between first or last result log appearance and correct submission compared to the rank of first or last appearance

To compare the two systems based on how fast result sets can be browsed and correct results can be found, we can look at two different metrics: The time delta between the *first* time the correct result appears in the results and submission time, and between the *last* time the correct result appears before submission and submission time. The first option gives us insights into cases where the correct result would have been already in the results, but the operator chose to reformulate the query, while the second option really tells us about the time it took to browse the final result set before submission. Both cases are shown in Fig. 18.

As can be seen from Table 1, vitrivr solved eight tasks, while vitrivr-VR only solved six, however, both plots in Fig. 18 only contain four data points for vitrivr-VR. What may appear like an error on first glance is actually another indicator of the deficiencies of the current data logging format. In the two cases of missing data, the correctly submitted segment never appeared as a direct result of any query formulated through vitrivr-VR and was most likely found as a result of the many result exploration options. In particular, what most likely led to the correct submission in these cases, was the use of the drawer view originating from a different segment of the same day as the correctly submitted segment. This theory is corroborated by the log of the vitrivr-VR interaction events, which show that the use of the drawer view was the last logged interaction before submission.

**Fig. 19:**
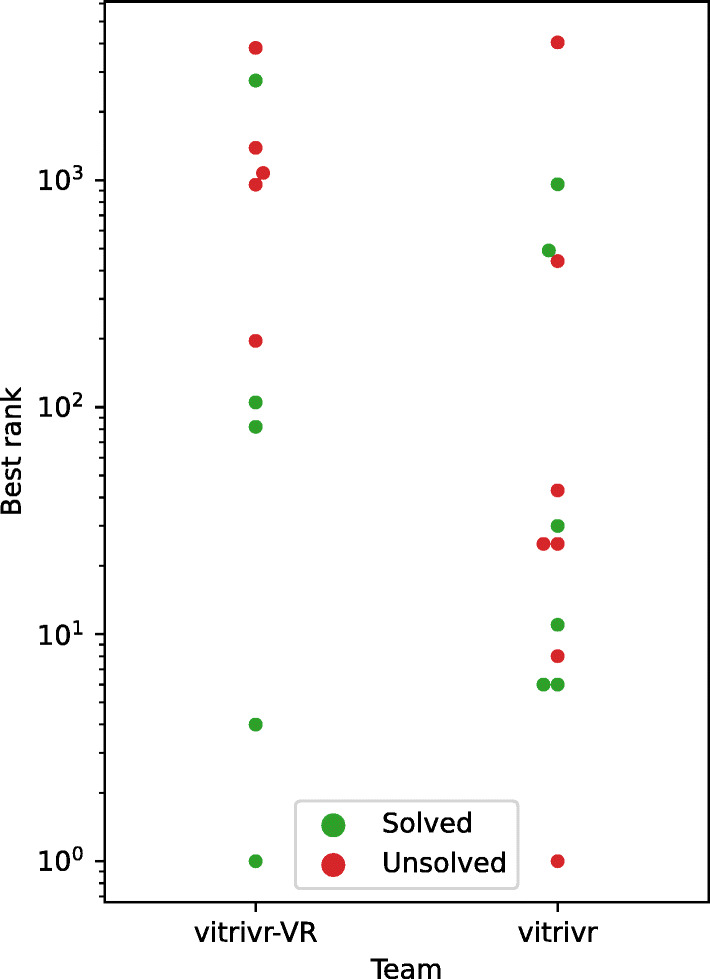
Best rank of any correct item per task and team. Red dots are operator browsing misses, i.e., the correct item would have been in the results, but did not get submitted

In Fig. 19, we show all cases where a correct item did appear in the result list, regardless of whether it was submitted or not. It is clearly visible in Fig. 19 that for both systems, there were operator errors, where a correct item would have been in the results, but was not submitted. While it is somewhat expected that for images which have a poor rank, an operator might not browse that far, there are a significant amount of browsing misses for vitrivr between ranks 1 and 100, which indicates operator errors as these are items that were most likely seen during browsing. Due to the low number of data points it is difficult to make statistically robust statements, but it appears that the queries of the vitrivr operator led to results with a higher precision than those of the vitrivr-VR operator, but that correct results may be easier to spot in vitrivr-VR than in vitrivr.

### Recommendations for data collection

Based on our experience and results, we highlight possible areas of improvement for the current methodology of LSC, both conceptually and for the current infrastructure.

#### Multiple independent participants

As previously mentioned, the current format for the LSC means that we only have data from one system instance available. Having more instances of the same system participating independently would allow us to perform different analyses, such as comparing inter-operator and inter-system differences as done in [27], and increase confidence and robustness in the results while using the same methodologies as done here.

#### One operator per system instance

The fact that the rules allow for multiple participants to be using the instance collaboratively, as long as there is only one operator means that some systems could have five people searching on the same screen and brainstorming for queries, while others could be using a single operator. This puts VR systems at a disadvantage, since they are more challenging to be used cooperatively, but also makes results analysis more challenging since there is an additional variable to be considered.

#### Data format

The current data format provided by the evaluation server only allows data analysis beyond that already provided as statistics calculated by the server after significant data integration effort. As of the version used at LSC,^13^ the evaluation server provides (i) a JSON file containing the most important information about an interactive retrieval competition event, such as the competing teams, the tasks that were used during the competition, and the submissions per task, and (ii) a CSV file containing a number of precomputed statistics resolved by team and task, such as the time until a correct submission was made.

While the evaluation server supports the logging of query results and interaction events, which was encouraged but not required at the LSC’21, this data is not contained within the standard set of analysis data provided by the server. This information is only accessible in the form of a complete log of all server events containing query results logs, interaction logs, submission logs, and task start and end events.

As this data, with the exception of submission logs, is only resolved by timestamp and session ID, a number of reconciliatory steps are required to connect it with the rest of the data and allow analysis to be performed. Two additional files are needed from the evaluation server maintainers: 1. the audit log file containing session logins resolved by username and timestamp, and 2. a file manually compiled by the evaluation server maintainers containing the mapping between usernames and user IDs. To import the data into a common schema session IDs must be resolved to usernames by matching login and logout time windows, usernames must be resolved to user IDs, query result and interaction logs must be resolved to tasks and users by matching task time windows and session IDs, and items in query result logs, which may be in any format, must be converted to the same format and matched with task targets. This reconciliation effort is additionally complicated through the mixed use of unlabeled server and client timestamps.

Based on these insights on the analysis data format, we make the following recommendations for future data collection: 1. specify and enforce a unified format both for data collected from participating systems, such as query result items, and for data logged by the server, such as timestamps, 2. link data appropriately on server side to ensure a consistent interpretation of analysis data across all individual team analyses, 3. provide all data in an appropriate format, such as CSV files or a relational database, and 4. document data sufficiently to prevent ambiguities and misinterpretations during analysis.

#### Collected data

At the current time, the data collected from different retrieval systems is sufficient only for very limited analyses. To be able to thoroughly analyze the performance of different systems and identify the highest performing user strategies, more data must be collected to reveal the user interactions and data pathways that led to successful submissions. With the current data collection strategy, which only specifies the collection of query result, basic interaction, and submission data, it is often impossible to determine which query led to a submission and what interactions were involved.

We make the following recommendations for data collection to enable more in-depth analysis in the future: 1. specify a unified format for the different interaction methods that can lead to a submission to allow the quantitative analysis of this data without post-hoc reconciliation or interpretation of logs, and 2. expand and formalize the data collection to ensure the necessary data required to trace the submission back to the first relevant interaction is collected for all systems

We are aware that these recommendations require significant research effort to adequately implement, but we believe that this is could greatly improve the depth of analysis possible for interactive retrieval competition data.

## Conclusion and future work

In this paper, we have compared and contrasted the retrieval and interaction approaches of two participating systems at LSC, vitrivr and vitrivr-VR. In addition to a careful description of the conceptual underpinnings of the systems, the different user interfaces have been described, with a special focus on the affordances of VR.

Our analysis of the result logs shows that, while the two systems perform similarly, with vitrivr performing slightly better, both systems have different strengths and weaknesses. The analysis clearly shows the need for improved data collection, in terms of the kind of data, the quantity, and the format. Multiple independent users per system, collection of data clearly linking submissions to operator interactions and queries, and the specification of a clear, formally defined schema for interaction and results logging would enable a vastly more robust analysis and would allow insights into the performance of systems and operators that are currently impossible to obtain.

LSC offers a great platform to gain insights through a holistic evaluation of end-to-end systems with users posing interactive queries in real-time. Participating with a VR system and a conventional retrieval system allows us to compare the opportunities and challenges of VR in a fair manner, as the shared retrieval backend eliminates the retrieval model from the comparison.

For future iterations of LSC, we plan to continue on our journey towards a general-purpose multimedia retrieval engine. Newer releases of the retrieval engine include, among others, a newer text-embedding model [37], new state-of-the-art visual-text co-embedding features such as CLIP [21], and a new OCR feature module [41].

One interesting avenue of research would be lifelog summarization, similar to approaches in video retrieval [2]. Especially in lifelogs, there is a lot of overlap between consecutive images, which means visually grouping similar shots or exploring different ways to ensure users can focus on browsing meaningfully different images and events.

## Footnotes

1 https://vitrivr.org

2 https://github.com/vitrivr/vitrivr-vr

3 Provided annotations by external APIs, e.g., OCR data and textual descriptions can be considered as metadata in this context.

4 If our data model would only consider the LSC dataset, we would not necessarily need the object abstraction, but as the model and systems are built for general purpose multimedia retrieval, we need to select an object abstraction.

5 The divisor can be configured based on the dataset and user preference, and was set to 100’000 for LSC 2021

6 https://www.typescriptlang.org/

7 https://angular.io/

8 https://github.com/mozilla/DeepSpeech

9 https://leafletjs.com/

10 https://www.openstreetmap.org

11 https://github.com/smeijer/leaflet-geosearch

12 Features are grouped by the backend into so-called *categories*, and technically results are returned per category. That implementation detail is not particularly relevant, as often categories only contain a single feature. Categories can also be thought of as hierarchical features.

13 v1.0.1,https://github.com/dres-dev/DRES/releases/tag/1.0.1

## Statements and Declarations

The code for the systems described in this paper is available open source at https://github.com/vitrivr/.

The logs analysed in this paper are available from corresponding authors or LSC organizers on reasonable request.
